# Clinical Characteristics of Patients with Endometrial Cancer and Adenomyosis

**DOI:** 10.3390/cancers13194918

**Published:** 2021-09-30

**Authors:** Paolo Casadio, Antonio Raffone, Manuela Maletta, Antonio Travaglino, Diego Raimondo, Ivano Raimondo, Angela Santoro, Roberto Paradisi, Gian Franco Zannoni, Antonio Mollo, Renato Seracchioli

**Affiliations:** 1 Division of Gynaecology and Human Reproduction Physiopathology, Department of Medical and Surgical Sciences (DIMEC), IRCCS Azienda Ospedaliero-Univeristaria di Bologna, S. Orsola Hospital, University of Bologna, Via Massarenti 13, 40138 Bologna, Italy; p.casadio@unibo.it (P.C.); manuela.maletta@studio.unibo.it (M.M.); roberto.paradisi@unibo.it (R.P.); renat.seracchioli@gmail.com (R.S.); 2 Gynecology and Obstetrics Unit, Department of Neuroscience, Reproductive Sciences and Dentistry, School of Medicine, University of Naples Federico II, 80131 Naples, Italy; 3 Pathology Unit, Department of Advanced Biomedical Sciences, School of Medicine, University of Naples Federico II, 80131 Naples, Italy; antonio.travaglino.ap@gmail.com; 4 Gynecologic and Obstetric Unit, Department of Medical, Surgical and Experimental Sciences, University of Sassari, 07100 Sassari, Italy; pwraimo@gmail.com; 5 School in Biomedical Sciences, University of Sassari, 07100 Sassari, Italy; 6 Gynecopathology and Breast Pathology Unit, Department of Woman’s Health Science, Agostino Gemelli University Polyclinic, 00168 Rome, Italy; angela.santoro@policlinicogemelli.it (A.S.); gianfranco.zannoni@unicatt.it (G.F.Z.); 7 Gynecology and Obstetrics Unit, Department of Medicine, Surgery and Dentistry “Schola Medica Salernitana”, University of Salerno, 84081 Baronissi, Italy; antmollo66@gmail.com

**Keywords:** endometrium, myometrium, tumor, carcinoma, malignancy, endometriosis

## Abstract

**Simple Summary:**

Endometrial cancer (EC) reportedly have a better prognosis in patients with coexistent adenomyosis. It is possible to hypothesize that this difference may be attributable to different clinical profiles. On this account, we aimed to define the clinical profile of endometrial cancer (EC) patients with and without adenomyosis through a systematic review and meta-analysis. We included 8 studies with 5681 patients and found that EC women with adenomyosis were less likely to be nulliparous than EC women without adenomyosis, while no significant differences were found with regard to age, BMI, and premenopausal status.

**Abstract:**

A better endometrial cancer (EC) prognosis in patients with coexistent adenomyosis has been reported. Unfortunately, it is still unclear if this better prognosis is related to a more favorable clinical profile of adenomyosis patients. We aimed to evaluate differences in the clinical profiles of EC patients with and without adenomyosis. A systematic review and meta-analysis was performed by searching seven electronics databases for all studies that allowed extraction of data about clinical characteristics in EC patients with and without adenomyosis. Clinical characteristics assessed were: age, Body Mass Index (BMI), premenopausal status, and nulliparity. Mean difference in mean ± standard deviation (SD) or odds ratio (OR) for clinical characteristics between EC patients with and without adenomyosis were calculated for each included study and as a pooled estimate, and graphically reported on forest plots with a 95% confidence interval (CI). The Z test was used for assessing the overall effect by considering a *p* value < 0.05 as significant. Overall, eight studies with 5681 patients were included in the qualitative analysis, and seven studies with 4366 patients in the quantitative analysis. Pooled mean difference in mean ± SD between EC women with and without adenomyosis was −1.19 (95% CI: −3.18 to 0.80; *p* = 0.24) for age, and 0.23 (95% CI: −0.62 to 1.07; *p* = 0.60) for BMI. When compared to EC women without adenomyosis, EC women with adenomyosis showed a pooled OR of 1.53 (95% CI: 0.92 to 2.54; *p* = 0.10) for premenopausal status, and of 0.60 (95% CI: 0.41 to 0.87; *p* = 0.007) for nulliparity. In conclusion, there are not significant differences in clinical characteristics between EC patients with and without adenomyosis, with the exception for nulliparity. Clinical features seem to not underlie the better EC prognosis of patients with adenomyosis compared to patients without adenomyosis.

## 1. Introduction

Endometrial cancer (EC) is the most common gynecologic malignancy, accounting for 25% of cancers in women worldwide [1,2].

The most important risk factor for the development of EC is obesity. Compared with other cancers, EC has the strongest association with obesity: while normal-weight women have a 3% lifetime risk of EC, the risk of cancer increases by more than 50% for every 5-unit BMI increase [3]. Other well-known risk factors are diseases associated with metabolic syndrome, including diabetes and polycystic ovary syndrome [3].

In addition, conditions characterized by excess of estrogen, such as hormone replacement with unopposed estrogen, nulliparity, early menarche, late onset menopause, and anovulatory conditions predispose women to EC. Otherwise, parity and oral contraceptive use provide protection against EC [3].

EC incidence has increased in the last decades, mainly due to an increased prevalence of such risk factors [4,5,6].

Adenomyosis represents one of the most frequent findings in the EC hysterectomy specimens [7,8]. It is a benign disease characterized by the migration of glands and stroma from the basal layer of the endometrium to the myometrium [9]. The exact pathogenesis is still poorly understood; since adenomyosis shows rapid growth, angiogenesis, and invasion of ectopic endometrial cells such as the malignant tumors, a possible link between adenomyosis and EC has been suggested [7].

The presence of co-existent adenomyosis has been reported to halve the risk of death in EC patients [10,11,12]. Unfortunately, it is still unclear if this better prognosis is related to a more favorable clinical profile (e.g., younger age or lower BMI) or a different underlying pathogenesis of EC in this subset of patients. In fact, some promoting factors are common to both diseases, such as unopposed hyper-estrogenic state, inflammatory milieu, and molecular features favoring cell proliferation and inflammation [7]. On the other hand, other clinical factors seem to differ. For example, EC is considered a cancer of the postmenopausal period (the sixth and seventh decades of life), while adenomyosis is mostly reported in women between 40 and 50 years [13]. Nulliparity is a risk factor for EC, while multiparity is for adenomyosis.

The aim of this study was to evaluate differences in the clinical profiles of EC patients with and without adenomyosis, in order to understand if clinical factors underlie a better EC prognosis in adenomyosis patients.

## 2. Materials and Methods

### 2.1. Study Protocol

Two authors independently performed each review step according to an a priori defined study protocol. Disagreements were discussed among all authors for solution. The whole study was reported following the Meta-analysis Of Observational Studies in Epidemiology (MOOSE) checklist [14].

### 2.2. Search Strategy and Study Selection

MEDLINE, Google Scholar, Scopus, Web of Science, ClinicalTrial.gov, Cochrane Library, and EMBASE were searched as electronic databases, for the period from their inception to September 2020. We performed several searches with several combinations of the following text words: “endometr*”, “adenomyosis”, “cancer”, “carcinoma”, “neoplas*”, “malignancy”, “tumour”, “tumor”, “myometr*”. The references list from each eligible article was also screened for any missed items.

We included all peer-reviewed studies that allowed extraction of data about clinical characteristics in EC patients with and without adenomyosis. A priori defined exclusion criteria were: case reports, literature review, and studies that performed a patient selection based on histological characteristics of EC or clinical features of patients. In fact, this latter criterion was necessary in order to avoid bias in the pooled data.

### 2.3. Assessment of the Risk of Bias within Studies

The risk of bias within studies was assessed by following the Methodological Index for Non-Randomized Studies (MINORS) [15]. Six applicable domains related to risk of bias were assessed in each included study: (1) aim (if the study aim was clearly stated); (2) patient selection (if patient selection was consecutive); (3) data collection (if an a priori defined study protocol was followed for data collection); (4) endpoints (if study outcomes were clearly reported); (5) endpoint assessment (if clinical characteristics of patients were assessed without bias); and (6) loss to follow-up (if patients with missing data about clinical characteristics were less than 5% of total study population).

Each included study was judged as “low risk”, “unclear risk”, or “high risk” of bias in each domain based on if data were “reported and adequate”, “not reported” or “reported but inadequate”, respectively.

### 2.4. Data Extraction

Data from the included studies were extracted without modification according to the PICO (Population, Intervention, Comparator, Outcomes) items [15,16,17,18].

“Population” of our study was patients with EC.

“Intervention” (or risk factor) was the diagnosis of adenomyosis.

“Comparator” was the absence of the diagnosis of adenomyosis.

“Outcome” was the clinical characteristics in EC patients. In particular, we assessed the following clinical characteristics: age, Body Mass Index (BMI), premenopausal status, and nulliparity.

We excluded from the quantitative analysis patients with EC arising from adenomyosis (EC-AIA) with regular eutopic endometrium [19], from two studies [20,21], and patients with EC and coexistent endometriosis or leiomyomas, from two studies [20,22].

### 2.5. Data Analysis

For continuous variables (age and BMI), mean difference in mean ± standard deviation (SD) between EC patients with and without adenomyosis was calculated for each included study and as a pooled estimate, and graphically reported on forest plots with a 95% confidence interval (CI).

For dichotomous variables (premenopausal status and nulliparity), the odds ratio (OR) between EC patients with and without adenomyosis was calculated for each included study and as a pooled estimate, and graphically reported on forest plots with 95% CI.

The random effect model of DerSimonian and Laird was used for all analyses, and the Z test was adopted to assess the overall effect by considering a *p* value < 0.05 as significant.

The inconsistency index I^2^ was used to judge statistical heterogeneity among the included studies. In particular, heterogeneity was judged as: null for I^2^ = 0%, minimal for 0 < I^2^ ≤ 25%, low for 25 < I^2^ ≤ 50%, moderate for 50 < I^2^ ≤ 75%, and high for I^2^ > 75%, as previously reported [17].

Review Manager 5.3 (Copenhagen: The Nordic Cochrane Centre, Cochrane Collaboration, 2014) was used as software for data analysis.

## 3. Results

### 3.1. Study Selection

Several searches identified 2410 studies. We excluded 1501 studies after duplicates removal, 883 after title screening, and 14 after abstracts screening. Finally, 8 studies remained after whole study assessment [11,12,20,21,22,23,24,25]. The study by Erkilinc et al. was excluded from the quantitative analysis because they randomly selected patients based on International Federation of Gynecology and Obstetrics (FIGO) grade of EC [11]. In fact, given the association between clinical characteristics and histological factors, this selection might affect pooled estimates of clinical characteristics (Supplementary Figure S1).

### 3.2. Included Studies and Study Population

All the included studies were designed as observational retrospective cohort studies, and assessed a total of 5681 women: 1322 (23.3%) with adenomyosis and 4359 (76.7%) without adenomyosis (Table 1 and Table 2).

The whole study population showed a mean age that ranged from 50.7 to 64.2 years, and a mean BMI that ranged from 25.2 to 35.8 kg/m^2^. Of the patients, 641 (33.3%) were premenopausal, and 473 (19.3%) nulliparous. Cancer antigen 125 (CA125) was >35 IU/L in 193 (20.8%) patients (Table 2). EC were non-endometrioid in 5.5% of cases, FIGO grade 3 in 15.2%, and FIGO stage II-IV in 17.4%. Moreover, EC showed deep myometrial infiltration in 20.9% of cases and LVSI in 18.8%.

### 3.3. Risk of Bias within Studies

All included studies were judged to be at “low risk” of bias in all domains, except the “patient selection” and “loss to follow up” domains. In particular, in the “patient selection” domain, two studies were judged at “unclear risk” of bias because they did not report if patient selection was consecutive [20,22], while one study was judged at high risk of bias since patient selection was based on FIGO grade of EC [11] (Supplementary Figure S2).

On the other hand, in the “loss to follow-up” domain, two studies were judged to be at “unclear risk” of bias because it was not possible to assess if patients with missing data about premenopausal status [21] and nulliparity [23] were less than 5% of the total study population, while another study was judged to be at high risk of bias because patients with missing data about nulliparity [12] were more than 5% of total sample (5.7% in “adenomyosis” group and 5% in “no adenomyosis” group).

### 3.4. Meta-Analysis

Data were extractable from three studies for age [12,20,21], and premenopausal status [20,21,24], two studies for BMI [12,25], and four studies for nulliparity [12,22,23,25].

In particular, we included in the quantitative analysis 909 patients (357 with adenomyosis and 552 without adenomyosis) for age, 921 (403 with adenomyosis and 518 without adenomyosis) for BMI, 1922 patients (236 with adenomyosis and 1686 without adenomyosis) for premenopausal status, and 2444 patients (996 with adenomyosis and 1448 without adenomyosis) for nulliparity.

**Figure 1:**
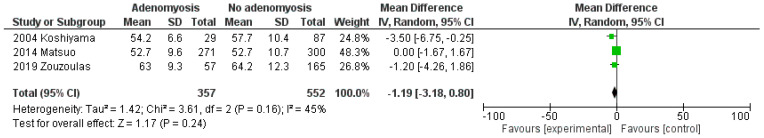
Forest plot of mean difference in mean ± standard deviation (SD) of age between EC patients with and without adenomyosis, for each included study and as pooled estimate.

**Figure 2:**
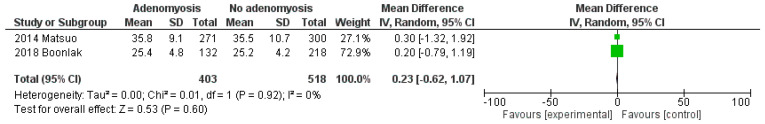
Forest plot of mean difference in mean ± standard deviation (SD) of body mass index between EC patients with and without adenomyosis, for each included study and as pooled estimate.

Pooled mean difference in mean ± SD between EC women with and without adenomyosis was −1.19 (95% CI: −3.18 to 0.80; *p* = 0.24; I^2^ = 45%) for age (Figure 1), and 0.23 (95% CI: −0.62 to 1.07; *p* = 0.60; I^2^ = 0%) for BMI (Figure 2).

**Figure 3:**
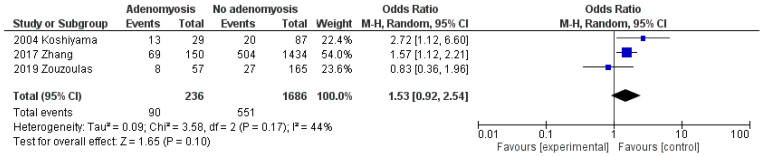
Forest plot of odds ratio for premenopausal status in endometrial cancer patients with adenomyosis compared to endometrial cancer patients without adenomyosis, for each included study and as pooled estimate.

**Figure 4:**
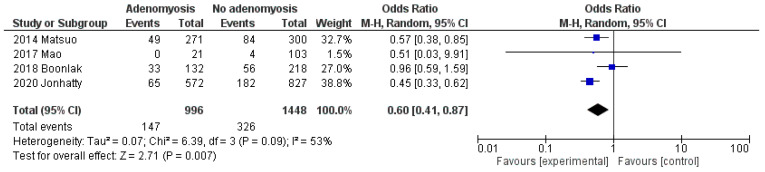
Forest plot of odds ratio for nulliparity in endometrial cancer patients with adenomyosis compared to endometrial cancer patients without adenomyosis, for each included study and as pooled estimate.

When compared to EC women without adenomyosis, EC women with adenomyosis showed a pooled OR of 1.53 (95% CI: 0.92 to 2.54; *p* = 0.10; I^2^ = 44%) for premenopausal status (Figure 3), and of 0.60 (95% CI: 0.41 to 0.87; *p* = 0.007; I^2^ = 53%) for nulliparity (Figure 4).

## 4. Discussion

### 4.1. Main Findings and Interpretation

This study shows that there are not significant differences in clinical characteristics between EC patients with and without adenomyosis, with the exception of nulliparity. In particular, EC patients without adenomyosis were more likely to be nulliparous than EC patients with adenomyosis.

Adenomyosis is a benign condition in which stroma and endometrial glands are found within the myometrial layer of the uterus, with a prevalence of 20–35% in women [8]. Adenomyosis typically presents with abnormal uterine bleeding, pelvic menstrual pain, and uterine enlargement at transvaginal ultrasound [8].

Although the association between adenomyosis and EC prevalence has not been proven [9], several authors have hypothesized a better EC prognosis in patients with underlying uterine adenomyosis [10,11,12]. However, the underlying factors are still unknown.

Some clinical features have been reported as prognostic factors of EC [26,27,28]. In particular, obesity has been associated with an increased risk of death due to EC [26]. Moreover, the prognosis of parous EC women has been reported to be significantly better than nulliparous ones [27]. Furthermore, age has also been shown to have a correlation with EC prognosis: as reported in a German population-based analysis, 5-year relative survival decreased from 90.0% in age group 15–49 years to 74.8% in age group 70 years [28]. However, to date, it is unclear if a different distribution of clinical features in EC patients with and without adenomyosis might explain the better prognosis in those with coexistent adenomyosis.

Our findings would indicate that clinical features do not explain the better EC prognosis of patients with adenomyosis compared to patients without adenomyosis.

In contrast, this better prognosis might be explained by a molecular hypothesis. In recent years, after The Cancer Genome Atlas (TCGA) Research Network findings and Proactive Molecular Risk Classifier for Endometrial Cancer (ProMisE) development, EC may be reclassified into four molecular prognostic groups: mismatch repair deficient, POLE-mutated, p53-mutated, and p53 wild-type [2]. POLE-mutated and p53 wild-type groups show a better prognosis [2]. Thus, a higher prevalence of these groups in EC patients with coexistent adenomyosis might explain the better prognosis of this subset of patients. It would be interesting to test this hypothesis, by assessing EC patients with coexistent adenomyosis thorough the ProMisE in future studies.

In our study, the only clinical characteristic that significantly differed between EC patients with and without adenomyosis was nulliparity. This finding is in accordance with the well-described association between adenomyosis and multiparity [29,30,31,32,33,34,35,36,37,38,39]. In particular, the frequency of adenomyosis appears directly associated with the number of pregnancies, with several explanations offered [40,41]. A first explanation might be that increasing parity is more likely to breach the endometria–myometrial junction, with the tissue injury and repair as the primary mechanism leading to glandular elements growing within the myometrium [42,43,44,45,46]. The same hypothesis might underlie pregnancy-associated uterine surgery, such as curettage [33], termination of pregnancy [47], and cesarean delivery [48,49,50]. Moreover, the same invasive nature of trophoblasts may also promote the invagination of the endometrium into the myometrium [40]. In addition, adenomyotic tissue shows a higher ratio of estrogen receptors compared to eutopic endometrium; thus, the increased hormone profile in pregnancy may also promote development of adenomyosis [40].

### 4.2. Strengths and Limitations

To the best of our knowledge, this may be the first systematic review and meta-analysis to assess differences in the clinical profiles of EC patients with and without adenomyosis. Our findings are supported by an overall good quality of the included studies, as shown by the risk of bias within studies assessment. On the other hand, as a limitation, all included studies had a retrospective design, and one study included in the quantitative analysis was judged at “high risk” of bias in one domain related to bias.

## 5. Conclusions

There are not significant differences in clinical characteristics between EC patients with and without adenomyosis, with the exception for nulliparity. Clinical features would not explain the better EC prognosis of patients with adenomyosis compared to patients without adenomyosis.

## Figures and Tables

**Table 1:** Characteristics of the included studies.

Study	Country	Setting	Type of Cohort	Period of Endometrial Cancer Diagnosis	Patient Selection
2004 Koshyama	Japan	Tenri Hospital and Himeji National Hospital	Retrospective cohort	1989–2001	Not specified
2014 Matsuo	USA	Los Angeles County Medical Center	Retrospective cohort	2000–2012	Consecutive
2017 Erkilinc	Turkey	University of Medical Sciences Tepecik Education and Research Hospital	Retrospective cohort	2007–2016	Consecutive
2017 Mao	China	Central Hospital of Lishui City, Lishui,	Retrospective cohort	2006–2013	Consecutive
2017 Zhang	China	Hebei general Hospital	Retrospective cohort	2008–2014	Consecutive
2018 Boonlak	Thailandia	Srinagarind Hospital	Retrospective cohort	2010–2016	Consecutive
2019 Zouzoulas	Greece	“Papageorgiou” Hospital, Thessaloniki	Retrospective cohort	2012–2017	Consecutive
2020 Jonhatty	Australia	Berghofor medical research institute	Retrospective cohort	Not specified	Not specified

**Table 2:** Clinical characteristics of endometrial cancer patients with and without adenomyosis.

Study	Total of Patients n		Adenomyosis n (%)	Age, [Years] Mean ± SD or Range)	BMI Mean ± SD	Premenopausal n (%)	Post-Menopausal n (%)	CA125 ± 35 IU/L n (%)	Nulliparity	Multiparity
2004 Koshiyama	116	Yes	29 (25)	54.2 ± 6.6	-	13 (45)	16 (55)	-	-	-
		No	87 (75)	57.7 ± 10.4	-	20 (23)	67 (77)	-	-	-
2014 Matsuo	571	Yes	271 (47.4)	52.7 ± 9.6	35.8 ± 9.1	-	-	59 (24.8)	60 (23.3)	197 (76.7)
		No	300 (52.5)	52.7 ± 10.7	35.5 ± 10.7	-	-	82 (30)	98 (34.4)	187 (65.6)
2017 Erkilinc	1242	Yes	80 (20)	56 ± 8.9	32.4 ± 7.0	-	-	-	-	-
		No	320 (80)	59 ± 24.8	32.9 ± 5.1	-	-	-	-	-
2017 Mao	127	Yes	24 (18.8)	50.7 (31–71)	-	-	9 (37.5)	3 (1.3)	0	-
		No	103 (81.1)	51.5 (31–72)	-	-	73 (70.9)	8 (7.8)	4 (3.9)	-
2017 Zhang	1584	Yes	150 (9.4)	53	27	69 (46)	81 (54)	-	-	-
		No	1434 (90.5)	55	26.6	504 (35.1)	930 (64.8)	-	-	-
2018 Boonlak	350	Yes	132 (37.7)	59 (36–80)	25.4 ± 4.8	-	-	-	33 (25)	99 (75)
		No	218 (62.2)	58 (31–84)	25.2 ± 4.2	-	-	-	56 (25.7)	162 (74)
2019 Zouzoulas	229	Yes	64 (27.9)	63.2 ± 9.4	-	-	56 (87.5)	10 (19.6)	-	-
		No	165 (72)	64.2 ± 12.3	-	-	138 (83.6)	31 (22.6)	-	-
2020 Jonhatty	1399	Yes	572 (40.8)	60.8 (31.9–80)	-	-	-	-	65 (11.4)	507 (88.6)
		No	827 (59.1)	61.6 (26.4–80)	-	-	-	-	182 (22.0)	645 (78)

SD: standard deviation; BMI: body mass index; CA125: cancer antigen 125.

## Supplementary Materials

The following are available online at https://www.mdpi.com/article/10.3390/cancers13194918/s1, Figure S1. Flow diagram of studies identified in the systematic review (Prisma template (Preferred Reporting Item for Systematic Reviews and Meta-analyses)), Figure S2. (a) Assessment of risk of bias. Summary of risk of bias for each study; plus sign: low risk of bias; minus sign: high risk of bias; question mark: unclear risk of bias. (b) Risk of bias graph about each risk of bias item presented as percentages across all included studies.
