# Supplementary figures and images for: Clinical Characteristics of Patients with Endometrial Cancer and Adenomyosis
Source: Cancers (Basel). 2021 Sep 30;13(19):4918. doi: 10.3390/cancers13194918 (PMC8508080; doi:10.3390/cancers13194918)

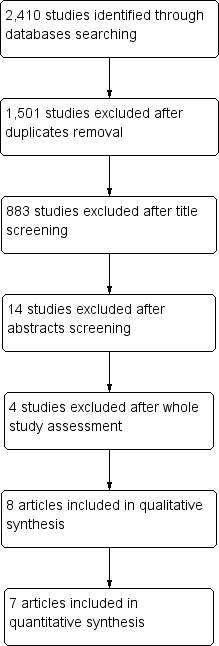

Supplement: Supplementary file 1 [file cancers-13-04918-s001.zip › Figure S1.png]

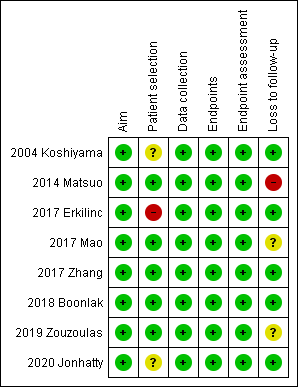

Supplement: Supplementary file 1 [file cancers-13-04918-s001.zip › Figure S2a.png]

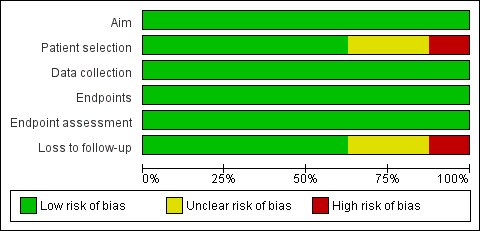

Supplement: Supplementary file 1 [file cancers-13-04918-s001.zip › Figure S2b.png]
